# Supplementary figures and images for: Malaria in Southern Venezuela: The hottest hotspot in Latin America
Source: PLoS Negl Trop Dis. 2021 Jan 25;15(1):e0008211. doi: 10.1371/journal.pntd.0008211 (PMC7861532; doi:10.1371/journal.pntd.0008211)

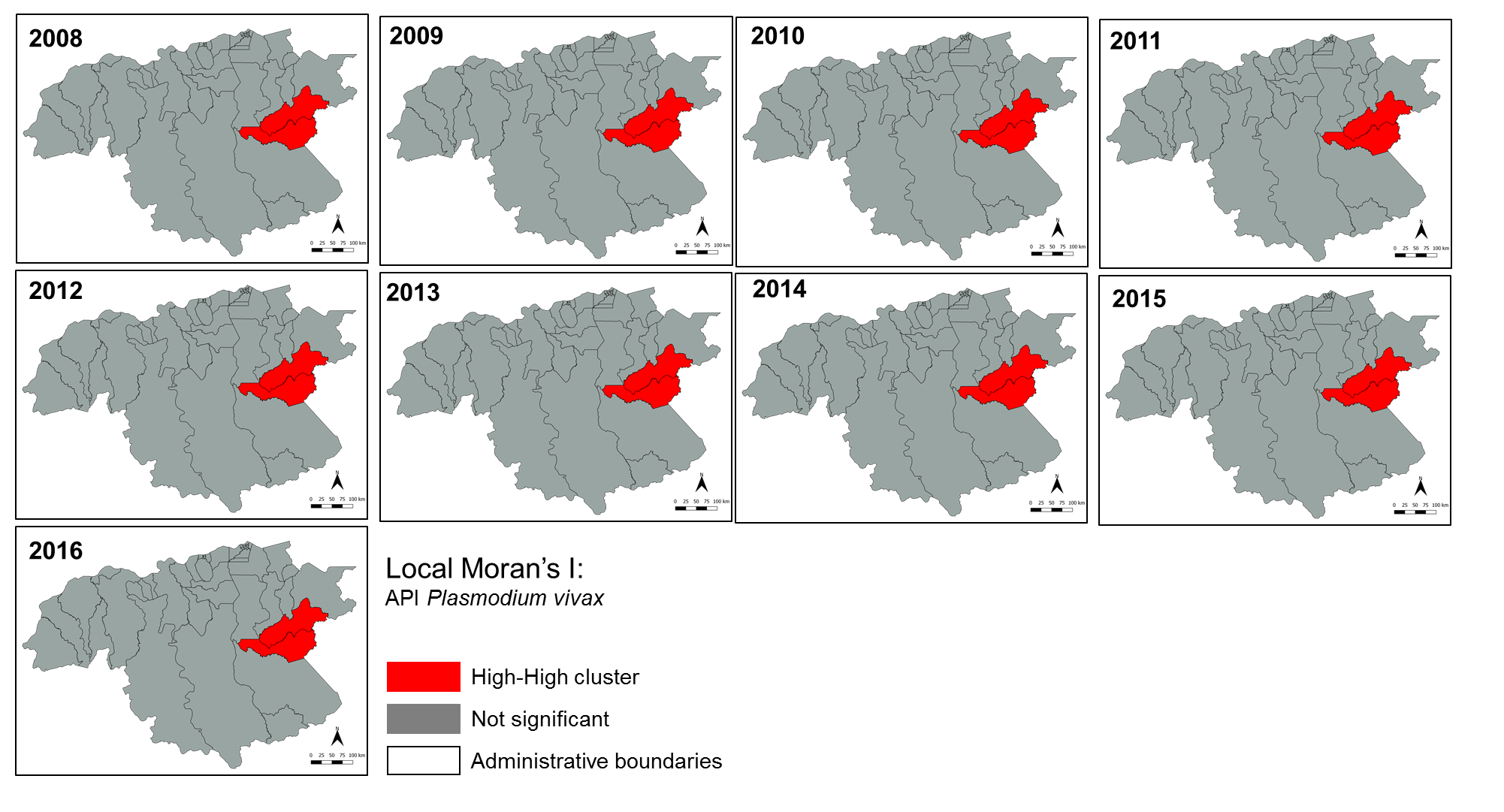

Supplement: S3 Fig — Maps were created with the Q-GIS software (https://www.qgis.org/es/site/) (TIF) [file pntd.0008211.s003.tif]

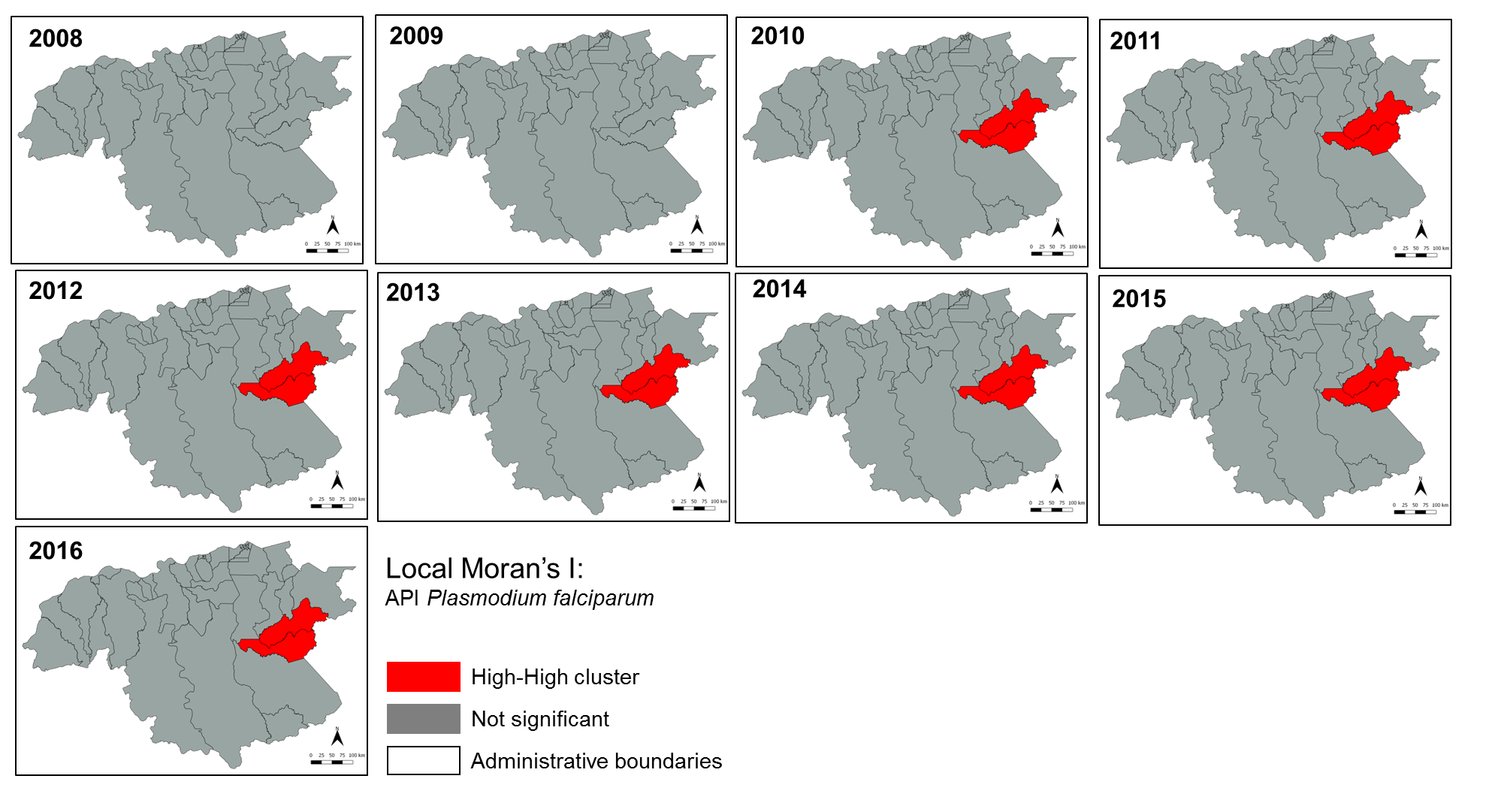

Supplement: S4 Fig — Maps were created with the Q-GIS software (https://www.qgis.org/es/site/). (TIF) [file pntd.0008211.s004.tif]

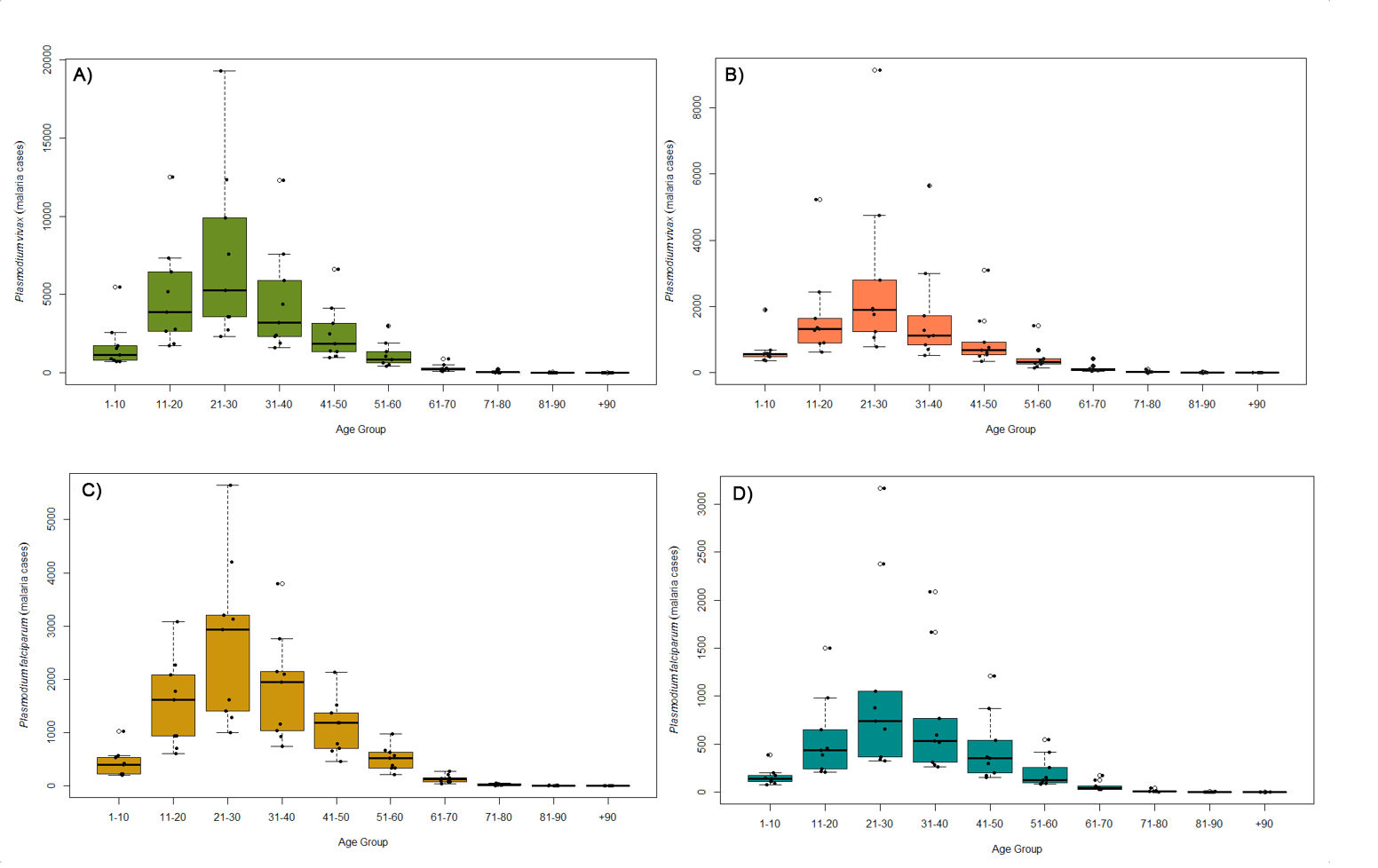

Supplement: S5 Fig — Age distribution of P. vivax (upper panel: A, B) and P. falciparum (lower panel: C, D) among malaria patients of San Isidro (left side: A, C) and Dalla Costa (right side: B, D) parishes (northeastern of Bolivar state) across the study period. (TIF) [file pntd.0008211.s005.tif]
