# Supplementary material for: Malaria in Southern Venezuela: The hottest hotspot in Latin America
Source: PLoS Negl Trop Dis. 2021 Jan 25;15(1):e0008211. doi: 10.1371/journal.pntd.0008211 (PMC7861532; doi:10.1371/journal.pntd.0008211)
Supplement: Attachment — Submitted filename: comments.pdf [file pntd.0008211.s006.pdf]

Major issues

Abstract

Several claims in the abstract are, in my point of view, unsupported and speculative. While it is fine to provide these hypotheses in the discussion section, I think that they should not have a prominent place in the abstract. Critically, I believe that several of the considerations the authors make are important but they do not arise primarily from the data and, as a result, would probably be a better fit with an opinion piece instead of a research article.

For example,

1) "We suggest that a source-sink pattern of Plasmodium sp. dispersal account for the re-emergence and progression of malaria transmission in the last 4 years across the country due to the internal migration of infected people to and from the hotspots and other malaria-prone ecosystems." The authors do not present any data on migration to support this. As a result, the depicted spatial patterns could be a result just of the collapse of the health system with a large increase in malaria incidence in areas more suitable for malaria and its vectors.
2) "We observe a similar pattern explaining the spillover of cases across international borders affecting neighboring countries." While the authors present some data from the Brazilian Ministry of Health, this is not the primary data that are the focus of their analysis.
3) "This study provides baseline epidemiological data and guidance for malaria control to further assess the dynamics of cross-border malaria, the role of asymptomatic carriers, drug-resistant evolution". There is little in this article about the role of asymptomatic carriers and drug-resistant evolution.

R0 calculations

Lines 111-120: As the authors point out in the discussion, there are a number of limitations associated with their calculation of R0. Therefore, it is not entirely clear to me that adding the R0 results contribute significantly to this article

Continuous surfaces

Line 138. "Finally, we used kriging, a local geostatistical interpolation method [29], to generate an estimated continuous surface from the scattered set of points (i.e., municipality centroids) to better capture the local spatial variation of malaria spread across the country during the 2014-2017 period". There are much better methods to estimate this continuous surface, such as GAMs (generalized additive models) and geospatial regression models.

Chi-square test

Lines 193-201: It would be useful to create a figure displaying the proportion of people in each occupation that were tested for malaria that obtained a positive test outcome (e.g., (#miners diagnosed with malaria)/(#miners tested)). The same could be done for age groups and gender. In my perspective, this would be more informative than a chi-square test because, given the sample size, this test is likely to detect very small deviations from the null hypothesis. These deviations, even if statistically significant, may or may not be epidemiologically significant.

Increase in population vs mining activities

The significance of the results in Fig. 7 depends on the size of the population at each locality. Can the clustering of localities > 1000 malaria cases be just an artifact of greater population in these areas?

Fig. 8. Perhaps the results shown here are again just an artifact of population size. In other words, tree cover lost (%) might just be a proxy for population density. In this case, perhaps it would be more accurate to state that: "Increase in malaria cases is associated with increased population in these malaria prone regions, most of which were attracted by the gold mining activities"

Forest loss vs mining

Line 133. "Additionally, to identify illegal mining operations in the study area, we obtained data on the deforested land cover distribution areas in the parishes classified as hotspots across the study period via Global Forest Watch". Is illegal mining always the reason for deforestation in this region? This is an implicit assumption in this article that may or may not be true.

#-----------------------

Other details

Lines 62-72: It would be nice if the map showed the names of the municipalities. That would allow the reader to follow the description of where is the state capital, where is the main urban center, etc. etc.

Line 119 "then estimated R0 as the calculus of the rate of". What do you mean by calculus? Derivative?

Line 129-130. "from the surveyed population for malaria in Bolivar state". Do you mean the people that were tested for malaria? I am assuming that these data arise from health facilities. If that is the case, I think that it is misleading to talk about these data as coming from a "survey".

Line 36 Clarify what you mean by “Route 10”. For example, replace “(Route 10)” by “(e.g., through the road to Brazil called Route 10)”.

Fig. 1 It is hard to see the river (it should be blue in the map but it is not) and to see route 10 (make line thicker)

Line 156. “sharp (exponential) and significant increase since 2014 ($R2 = 0.92$, $P < 0.05$).” Not sure what type of model was used here and that generate these results

Line 158: confusing phrase “Bolívar contributed about half (~47%) of the total cases in Venezuela during 2017, but this proportion represented from 60 to 80% in the previous years,”

Fig. 3 It is not possible to see the name of the parishes. As a result, it is hard to follow the description of the results in lines 169-175

“spatial heterogeneity was also detected for P. falciparum” Do you mean “there was also substantial spatial heterogeneity for P. falciparum”?

Supplementary Fig. 1: this is not a useful figure to me because it is influenced by the number of individuals (regardless of malaria status) in each age group. For example, if more people from the 21-30 age group are tested, it is more likely that we will see a greater number of cases in this age group, even if incidence per 1,000 in each age groups.

Line 203: what do you mean by “localities”? My understanding was that data were aggregated at the parish level. Are the data available at an even finer geographical scale?
