# Supplementary material for: Malaria in Southern Venezuela: The hottest hotspot in Latin America
Source: PLoS Negl Trop Dis. 2021 Jan 25;15(1):e0008211. doi: 10.1371/journal.pntd.0008211 (PMC7861532; doi:10.1371/journal.pntd.0008211)
Supplement: Attachment — Submitted filename: Rebuttal letter_Grillet et al. 2020.pdf [file pntd.0008211.s007.pdf]

UNIVERSIDAD CENTRAL DE VENEZUELA
FACULTAD DE CIENCIAS
INSTITUTO DE ZOOLOGIA Y ECOLOGIA TROPICAL
LABORATORIO DE BIOLOGIA DE VECTORES Y PARASITOS

Caracas July 25, 2020

Dr. Rhoel Ramos Dinglasan
Associate Editor
PLOS Neglected Tropical Diseases

Dr. Marcelo Ferreira
Deputy Editor
PLOS Neglected Tropical Diseases

Dear Dr. R. Ramos Dinglasan and Dr. M.U. Ferreira (Editors),

Please find a copy of the revised manuscript entitled: "**Malaria in Southern Venezuela: The Hottest Hotspot in Latin America**". This new version of the paper has taking into account most of the suggestions of the reviewer and the editor. The new manuscript comprises 28 pages of text and references, 7 figures with legends (included at the end of the text) and 2 Tables and 5 figures as Supplementary Material. You will see the point-by-point answers to all the corrections below.

All the contributing authors of the present manuscript have participated in the original study and they also concur with the resubmission of this new revision of the manuscript which is submitted by the corresponding author. Similarly, all the authors declare that they have not competing interests.

Sincerely

María-Eugenia Grillet, PhD
Full Professor
mariaeugenia.grillet@gmail.com

UNIVERSIDAD CENTRAL DE VENEZUELA
FACULTAD DE CIENCIAS
INSTITUTO DE ZOOLOGIA Y ECOLOGIA TROPICAL
LABORATORIO DE BIOLOGIA DE VECTORES Y PARASITOS

A point-by-point reply to the reviewer's comments

Reviewer #1: I believe that the methods employed in this article could be substantially improved. Specific comments are provided in the attached document.

Authors: We thank the reviewer for his comments and agree with his thoughtful suggestions. All comments and suggestions have been addressed and we believe they have greatly improved the description of the methods (see below).

Reviewer #2: The objectives are clearly outlined. However, in reading, there is some confusion as to what starting points represent hard data, and what are modeled estimates. The methods section could be improved with more clarity addressing this. Given the current situation there in Venezuela, for example, what are the pitfalls of using either MoH or even PAHO data? No concerns in re ethics or regulatory requirements. More generally, the main objective seems to be to identify the hotspots, yet there is a lot of text in the paper that does not really focus the reader's attention on this.

Authors: We thank the reviewer for his/her timely comments and agree that our initial version was prone for confusion. We have described in greater depth the methodology section. First, we have specified in the *Epidemiological and Sociodemographic Data* from the *Methods* section that this is a retrospective and descriptive study. The main objective was to describe demographic data and epidemiological profiles for all diagnosed individuals by the local health authorities and included in the local malaria database from the 2007-2017 period in the State of Bolivar (endemic region). We did not carry out malaria surveys. In addition, we expanded in some more detail about the nature, quality and characteristic of data used for this (methods) section in order to address the main and legitimate concern about reliability of the malaria data set. Now the text reads as (L98): *Historically, local health services in Venezuela have reported malaria cases detected by passive surveillance on a weekly basis according to geographic origin, and symptomatic cases. Malaria treatment is carried out for the malaria surveillance system, and the treatment provided free of charge. Case confirmation usually requires clinical and epidemiologic data (e.g., exposure history in endemic areas) plus a positive blood smear. The Venezuelan Ministry of Health (MofH) uninterruptedly has issued weekly and monthly epidemiological reports known as the "Boletín Epidemiológico" reporting on number of malaria cases, type of infection (parasite species), distribution per state, Municipality and Parish, distribution per age and sex, occupation of the patient, risk maps, and number of detected imported cases. At the country level, all reported malaria cases are notified via the National Notifiable Diseases Surveillance System (NDSS) by filling out a questionnaire containing relevant epidemiological inquires. Despite the Venezuela's health-care crisis, local malaria surveillance and data collection continued to occur and weekly bulletins were made available until 2017 and then interrupted since* [23].

Last, regarding the focus of the manuscript, we herein clarify that our main objective was to detect the hotspots; this has been corrected in the revised version of the manuscript. For example, see the new version of the Abstract.

Av. Paseo Los Ilustres, Los Chaguaramos, Caracas. Apartado postal 47058 - Caracas 1041-A.
Teléfonos: 58-212-6051404
E-mail: mariaeugenia.grillet@gmail.com

Reviewer #1: I do not think that the results are clearly presented and the figures could be substantially improved. Specific comments are provided in the attached document.

Authors: We have followed the recommendations (below) about this particular point.

Reviewer #2: The image quality in Figure 1 is poor. In figures 3 and 4, since no denominator is indicated, one assumes that what is illustrated is total number of cases; this is perhaps less useful to those not familiar with the population distribution in Venezuela than if the data were converted to incidence RATE.

Authors: Agree. We have improved the quality of Figure 1. Indeed, the maps included in Figure 3 and 4 (original version) relate to incidence rate (Annual parasite incidence: cases/population x 1,000). We have included and highlighted this in the legends of new Figures 3, 4, and S1 Fig, S2 Fig. Please note that based on overall recommendations from the reviewers we have reordered the figures and moved a number of them into supplementary material (overall, 5 S Figures).

Reviewer #2: Since the pattern stays pretty similar from year to year, would a single consensus map be more useful for the reader, with this more granular imaging relegated to supplementary figures? Similarly, figures 5 and 6, although highly illustrative, are also highly repetitive, and could be delegated to supplementary figures, with a representative year, perhaps the most recent, having a greater impact on the reader, with the additional mapping available for perusal if desired.

Authors: Agree. The revised version includes years 2007 and 2017 for each species, with figures 3 and 4 depicting the incidence rate (Figure 3) and hotspot results (Figure 4). Images showing the reminder of years for each species and descriptions have been moved to the supplementary material (5 S Figures). We think these may be of interest to the general readership and to further assist in visualizing the spatio-temporal change of malaria in that region.

Reviewer #1: Some of the conclusions are not well supported by the data they present. Specific comments are provided in the attached document.

Authors: We took note of this recommendation in the revised version.

Reviewer #2: The modeling itself appears sound. Some of the material in the discussion section reads more like background, and could perhaps be consolidated with that. Instead, a more thorough discussion of the other assumptions (including estimating the reliability of the starting data) would be most helpful. The discussion of r nought is quite good.

However, more discussion on the population migrations and their changing patterns over time would tie the paper together better as well. What about urbanization?

Authors: We thank the reviewer for the timely comments. We have emphasized in the revised version that this is a descriptive retrospective work. As for the spatial analyses (hotspots and risk map), we have incorporated all the details of the analyses including the required estimation on degree of uncertainty. As per recommendation of Reviewer #1 the R nought analysis has been removed.

We agree with the reviewer about the relevance in adding into the discussion those aspects related to migratory movement and patterns. However, specific and accurate data on internal migrations in the country are not currently available. We are presently working in a study design to address this issue in the near future. Still, many of our previously published observations (see references 5, 6, 7, 13, 17, 18, 20, 21, 42 and 43) as well as data published by other groups (e.g., 4) provide a well-documented snapshot of this situation. We have fully cited this on our manuscript.

Also, we would like to highlight that ever-increasing trends towards urbanization in malaria endemic areas of Venezuela is not directly related to internal population movements driven by mining given that given that these areas are located in very remote distant rural areas.

Reviewer #1: Specific comments are provided in the attached document.

Authors: Thanks

Reviewer #2: 1. Some of the methods/explanation of terms could be put in supplemental material –it's very educational, but it contributes to the MS length. 2. Some statements need to be softened. A good example is on line 239, where it might be better to either state that the decreasing vegetation is ASSUMED to be attributable to mining activities, or include a citation for this if you have it.

Authors: 1. Agree. We have kept the most details on the methods following this suggestion and we believe the length of the Methods section is now appropriate on this amended version.

2. Here we kindly disagree. Illegal gold mining and its associated deforestation have rapidly increased and expanded in southern Venezuela (particularly in the municipality of Sifontes, state of Bolívar) since 2009, which is documented in reference [28]. The text is now clearer about this evidence and reference.

Av. Paseo Los Ilustres, Los Chaguaramos, Caracas. Apartado postal 47058 - Caracas 1041-A.
Teléfonos: 58-212-6051404
E-mail: mariaeugenia.grillet@gmail.com

Reviewer #2: Very minor points are: Line 10; I think you mean to say "As in other malaria-endemic countries in SA, PV dominates, accounting for..." (I doubt that the numbers are exactly the same in all those other countries, which is what you are currently saying!)

Authors: Agree. This has been modified accordingly (L10).

line 37: I think you mean "resistance" rather than "resistant" (noun versus adjective)

Authors: Thanks. This has been corrected (L35).

Reviewer #1: (No Response)

Reviewer #2: Overall, the paper is a bit long for the content. There is repetition and excessive attention to some background concepts. Consider what is actually relevant to your methods and results, and, as mentioned above, what helps explain the identification of “hotspots”. For example, the mosquito information given does not adequately support the rather strong statement on lines 231-3; this statement should refer to your results being reported, not possible other work unless referenced. The climatic information provided, although it provides color, also seems more suited to another manuscript in which climactic factors are more addressed.

Authors: Agree and appreciate this suggestion. The paper is now shorter than its original version. Some text and background concepts have been reduced (mosquito information) or deleted (Ro analyses, climatic information of the study area, references associated). On those lines, the abstract (see below) as well as the overall text are now more centered on the description of the hotspot’s (main objective of the paper).

Major Issues (Reviewer #1)

Abstract

Several claims in the abstract are, in my point of view, unsupported and speculative. While it is fine to provide these hypotheses in the discussion section, I think that they should not have a prominent place in the abstract. Critically, I believe that several of the considerations the authors make are important but they do not arise primarily from the data and, as a result, would probably be a better fit with an opinion piece instead of a research article.

Av. Paseo Los Ilustres, Los Chaguaramos, Caracas. Apartado postal 47058 - Caracas 1041-A.
Teléfonos: 58-212-6051404
E-mail: mariaeugenia.grillet@gmail.com

For example,

1) "We suggest that a source-sink pattern of *Plasmodium* sp. dispersal account for the re-emergence and progression of malaria transmission in the last 4 years across the country due to the internal migration of infected people to and from the hotspots and other malaria-prone ecosystems." The authors do not present any data on migration to support this. As a result, the depicted spatial patterns could be a result just of the collapse of the health system with a large increase in malaria incidence in areas more suitable for malaria and its vectors.

2) "We observe a similar pattern explaining the spillover of cases across international borders affecting neighboring countries." While the authors present some data from the Brazilian Ministry of Health, this is not the primary data that are the focus of their analysis.

3) "This study provides baseline epidemiological data and guidance for malaria control to further assess the dynamics of cross-border malaria, the role of asymptomatic carriers, drug-resistant evolution". There is little in this article about the role of asymptomatic carriers and drug-resistant evolution.

Authors: Agree. We thank the reviewers for his/her relevant comments and suggestions. We have now reduced and focused the abstract on the main aim of this study as well as the whole text.

R0 calculations

Lines 111-120: As the authors point out in the discussion, there are a number of limitations associated with their calculation of R0. Therefore, it is not entirely clear to me that adding the R0 results contribute significantly to this article

Authors. Agree. We have removed the R0 analyses from this paper.

Continuous surfaces

Line 138. "Finally, we used kriging, a local geostatistical interpolation method [29], to generate an estimated continuous surface from the scattered set of points (i.e., municipality centroids) to better capture the local spatial variation of malaria spread across the country during the 2014-2017 period". There are much better methods to estimate this continuous surface, such as GAMs (generalized additive models) and geospatial regression models

Authors: Here we kindly disagree. Our main objective with the kriging analyses was to use the variable "annual malaria incidence" to show and describe (and visualize) the spatial pattern of the malaria in the country trying to minimize the error or variance as derived from the spatial covariance in the data (interpolation technique). A cross-validation was performed to evaluate the robustness of the model. Our aim was not to predict or explain the malaria in relation to other independent variables where the GAM approach would be better.

Chi-square test

Lines 193-201: It would be useful to create a figure displaying the proportion of people in each occupation that were tested for malaria that obtained a positive test outcome (e.g., (#miners diagnosed with malaria)/(#miners tested)). The same could be done for age groups and gender. In my perspective, this would be more informative than a chi-square test because, given the sample size, this test is likely to detect very small deviations from the null hypothesis. These deviations, even if statistically significant, may or may not be epidemiologically significant.

Authors: Thank you. We somehow disagree with the reviewer in this aspect. As we now note in this revised version, this is a retrospective and descriptive study of confirmed malaria cases from generated by the passive malaria surveillance (local health authorities). Thus, our objective was to describe the main demographic and epidemiological profiles of all individuals diagnosed with malaria (malaria database) during the studied period (2007-2017). Clarification: We did not carry out malaria field surveys. We believe that a frequency analyses is a good fit for this objective. As we mentioned before, we have now made clearer this aspect on the methods section. However, taking into account the suggestion of the reviewer, we have now incorporated two new tables (see Table 1 and 2) to the paper showing the total numbers of cases in each category of occupation, gender or age to better approach and depict the discussed results.

Increase in population vs mining activities
The significance of the results in Fig. 7 depends on the size of the population at each locality. Can the clustering of localities > 1000 malaria cases be just an artifact of greater population in these areas?

Authors: No. These localities and the region in general are rural low-density areas, and these are mainly localities that rely on mining driven economy. Denser localities located north of the Municipality do not exhibit higher malaria occurrence as these localities localized in mining deforested areas.

Fig. 8. Perhaps the results shown here are again just an artifact of population size. In other words, tree cover lost (%) might just be a proxy for population density. In this case, perhaps it would be more accurate to state that: "Increase in malaria cases is associated with increased population in these malaria prone regions, most of which were attracted by the gold mining activities"

Authors: Thanks for this suggestion. In the discussion we have addressed this issue as (L264): *"Thus, a hypothesis to be tested in future studies in southern Venezuela would be how forest fragmentation by mining activities may influence the ecology and vectorial capacity of both vector species regardless that the increase in malaria cases could be associated with increased population in these malaria prone regions, most of which are attracted by the gold mining activities."*

Forest loss vs mining
Line 133. "Additionally, to identify illegal mining operations in the study area, we obtained data on the deforested land cover distribution areas in the parishes classified as hotspots across the study period via Global Forest Watch". Is illegal mining always the reason for deforestation in this region? This is an implicit assumption in this article that may or may not be true.

Authors: Yes. We have made clearer this in the methods (L133) while including a reference (#29). See previous comments.

Other details
Lines 62-72: It would be nice if the map showed the names of the municipalities. That would allow the reader to follow the description of where is the state capital, where is the main urban center, etc. etc.

Authors: Thanks. It has been changed accordingly

Line 119 "then estimated R0 as the calculus of the rate of". What do you mean by calculus? Derivative?

Authors: This analysis has been removed of this revised manuscript

Line 129-130. "from the surveyed population for malaria in Bolivar state". Do you mean the people that were tested for malaria? I am assuming that these data arise from health facilities. If that is the case, I think that it is misleading to talk about these data as coming from a "survey".

Authors: Agree. We have explained that this is not a survey but a retrospective analysis (L110).

Av. Paseo Los Ilustres, Los Chaguaramos, Caracas. Apartado postal 47058 - Caracas 1041-A.
Teléfonos: 58-212-6051404
E-mail: mariaeugenia.grillet@gmail.com

Line 36 Clarify what you mean by "Route 10". For example, replace "(Route 10)" by "(e.g., through the road to Brazil called Route 10)".

Authors: Done.

Fig. 1 It is hard to see the river (it should be blue in the map but it is not) and to see route 10 (make line thicker)

Authors: This has been modified.

Line 156. "sharp (exponential) and significant increase since 2014 (R2 = 0.92, P < 0.05)." Not sure what type of model was used here and that generate these results

Authors: This is now specified in the revised text, see L151 (exponential model).

Line 158: confusing phrase "Bolívar contributed about half (~47%) of the total cases in Venezuela during 2017, but this proportion represented from 60 to 80% in the previous years,"

Authors: This has been edited for improved reading (L152).

Fig. 3 It is not possible to see the name of the parishes. As a result, it is hard to follow the description of the results in lines 169-175

Authors: This has been modified.

"spatial heterogeneity was also detected for *P. falciparum*" Do you mean "there was also substantial spatial heterogeneity for *P. falciparum*"?

Authors: Yes. This has been edited and now reads more fluently (L176).

Supplementary Fig. 1: this is not a useful figure to me because it is influenced by the number of individuals (regardless of malaria status) in each age group. For example, if more people from the 21-30 age group are tested, it is more likely that we will see a greater number of cases in this age group, even if incidence per 1,000 in each age groups.

Authors: We kindly disagree. As we explained before, this is a retrospective and descriptive study of the confirmed cases of malaria (passive surveillance system); thus, our objective was to describe the demographic and epidemiological profiles of all individuals diagnosed with malaria (malaria database) during the studied period (2007-2017). We did not carry out malaria field surveys. As we mentioned before, we have made clearer this aspect in the section of methods of the new version. In addition, we have supported these analyses with two new tables (Table 1 and 2) showing the total numbers of cases in each category of occupation, gender or age for a better visualization of the discussed results as it is requested for this reviewer. We want to keep the Figure (Now S5 Fig) in the supplementary material for a better visualization of the discussed results.

Line 203: what do you mean by "localities"? My understanding was that data were aggregated at the parish level. Are the data available at an even finer geographical scale?

Authors: In Figure 7 -original version- we sketched a landscape close-up to show the concentration of localities with malaria in that parish. This is explained in the figure legend. Although most of the analyses in the manuscript are at the parish level, here, we just wanted to display that result.

Av. Paseo Los Ilustres, Los Chaguaramos, Caracas. Apartado postal 47058 - Caracas 1041-A.
Teléfonos: 58-212-6051404
E-mail: mariaeugenia.grillet@gmail.com
