# Supplementary material for: Malaria in Southern Venezuela: The hottest hotspot in Latin America
Source: PLoS Negl Trop Dis. 2021 Jan 25;15(1):e0008211. doi: 10.1371/journal.pntd.0008211 (PMC7861532; doi:10.1371/journal.pntd.0008211)
Supplement: Attachment — Submitted filename: Point_by_Point_ letter_Grillet et al. 2020_Malaria_Hotspot.pdf [file pntd.0008211.s008.pdf]

UNIVERSIDAD CENTRAL DE VENEZUELA
FACULTAD DE CIENCIAS
INSTITUTO DE ZOOLOGIA Y ECOLOGIA TROPICAL
LABORATORIO DE BIOLOGIA DE VECTORES Y PARASITOS

A point-by-point reply to the reviewer's comments

(Please, see all these corrections in the copy with tracked changes since the number or identification of each line can be different in the clean version)

Reviewer #4:

-lines 140-145: Can you explain this analysis in more detail? Were the expected frequencies based on each parish?

Authors: It is explained now (see L160-171).

No, it is based on Municipality as it reads now in the text (L160): *Finally, we used kriging, a local geostatistical interpolation method [30], to generate an estimated continuous surface from the scattered set of points (i.e., municipality centroids) with z-values (i.e., annual parasite incidence in each municipality) to better capture the local spatial variation of malaria spread (spatial risk diffusion map) across the country during the 2014-2017 period*

-The maps and figures are very nice. In Figure 1, the name label for the civil parish overlaps the location of the capitol city – can this be changed? Also, in Figure 1, both the stare and the triangle are labeled at capital cities. For Figures 3 and 4, it does not seem that the civil parish labels can be read; it might be better to remove these names.

Authors: Maps and Figures were changed accordingly

-line 173: Is this p-value the result of an analysis for trend? This analysis should be described in the methods section.

Authors: Thanks for this comment. We added a brief sentence about this analysis in the methodology (L157): *"In order to determine if malaria cases exhibited an increasing trend (distinguished from random behavior) along the years or along the temporal cover tree loss, the best fit line (curve fitting approach) to the time series was applied to aid interpretation of data".*

-lines 174-178: "Bolivar contributed … southeastern region." This sentence is complicated/confusing – do you mean that more than half of the cases in Venezuela were from Bolivar during the time period?

Authors: Thanks. We changed it for a better reading.

-lines 179, 181, 182: The descriptive analyses (mean ratio calculations, trend tests) should also be in the methods section

Authors: Thanks for this comment. We added a brief sentence about this in the methodology (L157): *"In order to determine if malaria cases exhibited an increasing trend (distinguished from random behavior) along the years or along the temporal cover tree loss, the best fit line (curve fitting approach) to the time series was applied to aid interpretation of data".* L147: *The chi-squared test allowed us to determine whether there were significant differences between the expected frequencies and the observed frequencies for one or more categories".*

Av. Paseo Los Ilustres, Los Chaguaramos, Caracas. Apartado postal 47058 - Caracas 1041-A.
Teléfonos: 58-212-6051404
E-mail: mariaeugenia.grillet@gmail.com

-Figure 6: The lines in the figure clearly represent some analysis (correlation or regression) between the data, but the analysis is not described in the methods section

Authors: Thanks. It is already in the method section: (L157): *"In order to determine if malaria cases exhibited an increasing trend (distinguished from random behavior) along the years or along the temporal cover tree loss, the best fit line (curve fitting approach) to the time series was applied to aid interpretation of data".*

-Figure 7: What is the unit of measurement? Are these incidence rates?

Authors: Yes, it is the annual parasite incidence (API). We included that correction in the text and legend of the Figure 7.

(L160): *Finally, we used kriging, a local geostatistical interpolation method [30], to generate an estimated continuous surface from the scattered set of points (i.e., municipality centroids) with z-values (i.e., annual parasite incidence in each municipality) to better capture the local spatial variation of malaria spread (spatial risk diffusion map) across the country during the 2014-2017 period*

Fig 7. "*Spatial prediction maps (Venezuela malaria risk) for the 2014 (a), 2015 (b), 2016 (c) and 2017 (d) years derived from the ordinary gaussian kriging interpolation model of the annual parasite incidence (API). Note Year 2017, data available only through Epidemiology Week 21"*.

-There is no discussion of analysis limitations

Authors: We really appreciate this suggestion. We have added a text at the end of the manuscript (L393) and end of the Discussion section

Limitation of the study
The main limitations of this study are related to the nature of passive malaria surveillance data and the lack of local demographic information to standardize the analysis of occupational risk. Sub-reporting of malaria incidence is presumed due to a limited diagnostic capacity of the parasite detection in the region and the whole country as a result of the Venezuelan Health crisis. The lack of completeness of the surveillance systems was also a critical issue when we analyzed the 2017 malaria data. Both facts underestimate the total number of malaria infections; however, incidence data analyzed still gave us insights about the upward trend of malaria transmission and the localization of the main source (hotpots) of P. vivax and P. falciparum cases in Venezuela in the recent years as suggested by earlier studies [7,13,21]. Similarly, the gold mining as a significant disease social driver, and the land-use change (deforestation) as an important local environmental associated proxy of local malaria increase were such strong patterns that they emerged even with this limited data. The current findings support observations of our previous work [5-7, 12, 13, 17, 19, 21, 22, 42, 43]; nevertheless, future studies will be needed to further confirm these patterns and address the main questions emerged in this study.

We also added in the conclusion a segment in regard to this issue (L416): *Malaria surveillance and reporting have been particularly affected by Venezuela's healthcare crisis since mid-2017 onwards [7]. Hence, to recover and strengthen this surveillance system is a necessary step to control malaria in Venezuela.*

Av. Paseo Los Ilustres, Los Chaguaramos, Caracas. Apartado postal 47058 - Caracas 1041-A.
Teléfonos: 58-212-6051404
E-mail: mariaeugenia.grillet@gmail.com

-line 298-302: "Thus, a … mining activities". This sentence is complicated/too long. I suggest you split it into two sentences.

Authors: Thanks. We did split the sentence and now it reads as (L313): "*Thus, a hypothesis to be tested in future studies would be how forest fragmentation by mining activities may influence the ecology and vectorial capacity of both vector species. This ecological mechanism should be addressed regardless that the increase in malaria in the area be the result of increased population attracted by the gold mining activities"*

-line 313-314: Can you clarify – these migrants reintroduce malaria parasites when they return to other parts of Venezuela from the mines? Do these workers come to the gold mines to work and then return home (or make multiple trips)?

Authors: We modified the paragraph for a better understanding (L339): *"As a result, migration within the country has increased towards southeastern Venezuela (Sifontes Municipality) where gold mining activities are clustered and where malaria hotspots have been identified [44]. Highly mobile human populations migrate from different regions of the country to these mining areas in search of economic opportunities. Some of these internal migrants return back to the region of origin where viable anopheles vector populations exist, reintroducing malaria to areas where autochthonous transmission had been previously eliminated leading to a shift in the epidemiology of this disease [4,5,7]."*

-line 347: What is SP?

Authors: It is sulfadoxine/pyrimethamine, a combination treatment for malaria. But we eliminated it from the text.

Reviewer #2: (Minor changes in the text)
Line 3 'especially so' would read better than 'even so'.

Authors: It was changed

Line 23 comma after 'healthcare system'

Authors: It was added

Line 44 comma after 'neighboring countries'

Authors: It was included

Line 47 movements is plural so it should be "serve"

Authors: Corrected

Line 76 comma after both 'Venezuela' and 'America'

Authors: Included

Line 281, comma after [37]

Authors: Included

Line 285 meaning of this sentence is not entirely clear; is the case clustering or the deforestation the consequence of gold mining activity? Please rewrite this sentence for clarity.

Authors: Thanks for this suggestion. We have modified the sentence for a better reading. From L298-316, now it reads as:
"*First, we found that areas deforested by gold mining had more clusters of Plasmodium cases compared to areas nearby. Secondly, both P. vivax and P. falciparum increased in incidence (4-8-fold) over time in those regions along with a concomitant decrease of vegetation cover (3-6-fold) resulted from such mining activities. Finally, our results reporting age and sex patterns within the malaria patients of the region are compatible with the occupation as a factor determining the greater and prolonged exposure to vector bites of young males in the mining fields compared to girls and women. Illegal gold mining and the associated deforestation have rapidly increased and expanded in southern Venezuela since 2009 [29], particularly in the municipality of Sifontes, state of Bolívar. Changes in land cover patterns resulting from deforestation can promote the emergence of larval habitats for these Nyssorhynchus (also known as Anopheles) vectors, thereby increasing mosquito abundance [38], vector-host contact [39], survival [40] and consequently transmission risk [41], especially in human settlements located near the forest fringe. Earlier studies in our study area have indicated that the most productive breeding site types for Ny. albitarsis s.l. and to a lesser degree for Ny. darlingi, are abandoned open lagoons or mining dug-outs left after clearing vegetation [42, 43]. Thus, a hypothesis to be tested in future studies would be how forest fragmentation by mining activities may influence the ecology and vectorial capacity of both vector species. This ecological mechanism should be addressed regardless that the increase in malaria in the area be the result of increased population attracted by the gold mining activities".*

Line 290 should discuss that the perpetrators of illegal gold mining would be expected to be largely young males, so that this association might be related to transmission exposure rather than any kind of independent "other".

Authors: We agree with the reviewer, we added in the previously mentioned paragraph: "*Finally, our results reporting age and sex patterns within the malaria patients of the region are compatible with the occupation as a factor determining the greater and prolonged exposure to vector bites of young males in the mining fields compared to girls and women*".

Also, line 290, the sentence beginning with 'Illegal' seems like the start of a new paragraph, since it signals a switch to discussion of the vector.

Authors: Agree. We modified. See previous paragraph, from L298-316.

Line 349 comma after 'strains'

Av. Paseo Los Ilustres, Los Chaguaramos, Caracas. Apartado postal 47058 - Caracas 1041-A.
Teléfonos: 58-212-6051404
E-mail: mariaeugenia.grillet@gmail.com

Authors: Included

Line 350 the word 'response' should be inserted after 'artemesinin', perhaps with a hyphen linking the two

Authors: It was modified accordingly.

Figure 1; the labeling of the individual region names, and of the color key, is not publication quality. Figures 3 and 4; as for Figure 1

Authors: Figures were modified accordingly

Figure 5; the key is not as informative as the legend. A preferable key would say 'localities with <1000 cases' etc.

Authors: The key was modified according to the suggestion of the reviewer. The key now specifies localities with > and < 1,000 cases.

Figure 7; neither the figure itself nor the legend indicates the units, presumably cases, but per what denominator?

Authors: The legend is clearer now. The variable used by running the model was annual parasite incidence (API).

Reviewer #4:

-line 2: change "the last" to "recent"

Authors: Done

-line 3: change "even so" to "particularly"

Authors: Done

-line 5: remove "largely" (it is redundant)

Authors: It has been removed

-line 46: change "movements" to "movement"

Authors: It has been changed

-line 61: change "malaria resilience" to "the resilience of malaria"

Authors: It has been changed

-line 76: put a comma after “Venezuela”

Authors: It has been done

-lines 76, 80, 81: format “Km2” as “km²”

Authors: It has been changed

-lines 90, 91: change “exhibits lacks of seasonality” to “does not exhibit a seasonal pattern”

Authors: It has been changed

-lines 124-125: change “and then” to “but have been”

Authors: It has been changed

-line 128: health should be capitalized

Authors: It has been changed

-line 150: remove “The”

Authors: It has been removed

-line 151: change “the last” to “recent”

Authors: It has been changed

-line 199: remove the comma

Authors: It has been removed

-line 201: remove “also”

Authors: It has been removed

-Table 1: the parish is labeled “San Ignacio” but the text says “San Isidro”

Authors: Thanks. It has been corrected, it is San Isidro

-line 240: define “Ha”

Authors: It has been defined

-line 280: change “sum factors” to “sum of factors”

Authors: It has been corrected

Reviewer #2: There is still not a short discussion of the strengths and pitfalls of reliance on the source data; please include a sentence or two, and perhaps a reference, on the utility and reliability of this data.

Authors: Agree. We really appreciate this suggestion. We have added a text at the end of the manuscript (L393) and end of the Discussion section

Limitation of the study
The main limitations of this study are related to the nature of passive malaria surveillance data and the lack of local demographic information to standardize the analysis of occupational risk. Sub-reporting of malaria incidence is presumed due to a limited diagnostic capacity of the parasite detection in the region and the whole country as a result of the Venezuelan Health crisis. The lack of completeness of the surveillance systems was also a critical issue when we analyzed the 2017 malaria data. Both facts underestimate the total number of malaria infections; however, incidence data analyzed still gave us insights about the upward trend of malaria transmission and the localization of the main source (hotpots) of P. vivax and P. falciparum cases in Venezuela in the recent years as suggested by earlier studies [7,13,21]. Similarly, the gold mining as a significant disease social driver, and the land-use change (deforestation) as an important local environmental associated proxy of local malaria increase were such strong patterns that they emerged even with this limited data. The current findings support observations of our previous work [5-7, 12, 13, 17, 19, 21, 22, 42, 43]; nevertheless, future studies will be needed to further confirm these patterns and address the main questions emerged in this study.

We also added in the conclusion a segment in regard to this issue (L416): *Malaria surveillance and reporting have been particularly affected by Venezuela's healthcare crisis since mid-2017 onwards [7]. Hence, to recover and strengthen this surveillance system is a necessary step to control malaria in Venezuela.*

Reviewer #4:
-For the analysis of occupations, gender, and age structure among cases in hotspots (Tables 1 and 2), there needs to be additional detail in the methods section. It seems that the analysis is comparing the distribution of cases among categories within each parish. The link between occupation, gender, age, and malaria infection would be more convincing if the authors performed something like an indirect standardization. It is important to account for the underlying populations in these parishes – if 60% of the people living in San Isidro work in gold mining, we would not be surprised if 60% of people with malaria are gold miners – maybe malaria and gold mining are unrelated and San Isidro just has lots of people who work in mining. If malaria is truly related to gold mining, we first must account for San Isidro having many gold miners living there. If there are more gold miners with malaria than we would expect (given the high number of miners in the parish), then we can conclude that there is an overrepresentation of gold miners among malaria cases.

Authors: We really appreciate this comment. Unfortunately, there is not available information about the population or number of people per occupation at that scale (parish level or municipality level). This is a main limitation of our study. We have highlighted that issue in the *limitation of the study section* we added:

The main limitations of this study are related to the nature of passive malaria surveillance data and the lack of local demographic information to standardize the analysis of occupational risk. Sub-reporting of malaria incidence is presumed due to a limited diagnostic capacity of the parasite detection in the region and the whole country as a result of the Venezuelan Health crisis. The lack of completeness of the surveillance systems was also a critical issue when we analyzed the 2017 malaria data. Both facts underestimate the total number of malaria infections; however, incidence data analyzed still gave us insights about the upward trend of malaria transmission and the localization of the main source (hotpots) of P. vivax and P. falciparum cases in Venezuela in the recent years as suggested by earlier studies [7,13,21]. Similarly, the gold mining as a significant disease social driver, and the land-use change (deforestation) as an important local environmental associated proxy of local malaria increase were such strong patterns that they emerged even with this limited data. The current findings support observations of our previous work [5-7, 12, 13, 17, 19, 21, 22, 42, 43]; nevertheless, future studies will be needed to further confirm these patterns and address the main questions emerged in this study.

-Can you provide some background information (a couple sentences) on the link between deforestation and gold mining and malaria in the introduction/background section?

Authors: We included a new segment in the Background section (L39) that reads as: "Gold mining has been associated with the high malaria incidence in specific municipalities of this region where gold miners seem to account for an estimated 47% to 80% of malaria cases [4, 5, 13]. Clearing forest for mining activities is observed to create favorable conditions for main vector malaria species in this area [5, 7, 13]".

-Can you discuss the limitations of only having data from EW 1-21 in 2017 for the spatial prediction maps?

Authors: We mentioned that limitation in the new paragraph (see bold text below):

"*The main limitations of this study are related to the nature of passive malaria surveillance data and the lack of local demographic information to standardize the analysis of occupational risk.* ***Sub-reporting of malaria incidence is presumed due to a limited diagnostic capacity of the parasite detection in the region and the whole country as a result of the Venezuelan Health crisis. The lack of completeness of the surveillance systems was also a critical issue when we analyzed the 2017 malaria data. Both facts underestimate the total number of malaria infections;*** *however, incidence data analyzed still gave us insights about the upward trend of malaria transmission and the localization of the main source (hotpots) of P. vivax and P. falciparum cases in Venezuela in the recent years as suggested by earlier studies [7,13,21]. Similarly, the gold mining as a significant disease social driver, and the land-use change (deforestation) as an important local environmental associated proxy of local malaria increase were such strong patterns that they emerged even with this limited data. The current findings support observations of our previous work [5-7, 12, 13, 17, 19, 21, 22, 42, 43]; nevertheless, future studies will be needed to further confirm these patterns and address the main questions emerged in this study.*

We also added in the conclusion a segment in regard to this issue (L416**):** ***Malaria surveillance and reporting have been particularly affected by Venezuela's healthcare crisis since mid-2017 onwards*** *[7]. Hence, to recover and strengthen this surveillance system is a necessary step to control malaria in Venezuela.*

Av. Paseo Los Ilustres, Los Chaguaramos, Caracas. Apartado postal 47058 - Caracas 1041-A.
Teléfonos: 58-212-6051404
E-mail: mariaeugenia.grillet@gmail.com
